# National HIV Testing Day — June 27, 2017

**DOI:** 10.15585/mmwr.mm6624a1

**Published:** 2017-06-23

National HIV Testing Day, June 27, highlights the importance of testing in detecting, treating, and preventing human immunodeficiency virus (HIV) infection. Awareness of HIV infection through HIV testing is the first step to prevention, health care, and social services that improve quality of life and length of survival (*1*).

CDC’s National HIV Behavioral Surveillance (NHBS) monitors behaviors among populations at risk for acquiring or transmitting HIV infection. Recent NHBS data indicate that persons at risk for HIV infection who had ever received testing for HIV are testing at shorter intervals than in the past (*2*). The average interval in months between two successive HIV tests decreased from 21.1 in 2010 to 19.9 in 2013 among heterosexuals at increased risk for HIV, from 10.5 in 2009 to 7.7 in 2014 among men who have sex with men, and from 14.4 in 2009 to 11.5 in 2015 among persons who inject drugs.

Additional information on National HIV Testing Day is available at https://www.cdc.gov/features/HIVtesting. Basic testing information for consumers is available at https://www.cdc.gov/hiv/basics/testing.html. Additional information on HIV testing for health professionals is available at https://www.cdc.gov/hiv/testing. CDC’s guidelines for HIV testing of serum and plasma specimens is available at https://www.cdc.gov/hiv/guidelines/testing.html.

## References

[1] CDC. Vital Signs: HIV diagnosis, care, and treatment among persons living with HIV—United States, 2011. MMWR Morb Mortal Wkly Rep 2014;63:1113–7. 25426654 PMC5779517

[2] An Q, Song R, Finlayson TJ, Wejnert C, Paz-Bailey G; NHBS Study Group. Estimated HIV inter-test interval among people at high risk for HIV infection in the US. Am J Prev Med 2017. http://www.sciencedirect.com/science/article/pii/S0749379717301435 10.1016/j.amepre.2017.02.009 28336355

